# Evaluation of the Serological Baseline Values of Broiler Chickens Jointly Vaccinated with Infectious Bronchitis H + 120 and GI-13 Preparations Under Field Conditions

**DOI:** 10.3390/ani16050807

**Published:** 2026-03-05

**Authors:** Marcin Śmiałek, Joanna Kowalczyk

**Affiliations:** 1 Department of Poultry Diseases, Faculty of Veterinary Medicine, University of Warmia and Mazury, 10-719 Olsztyn, Poland; joanna.kowalczyk@uwm.edu.pl; 2 SLW BIOLAB, ul. Grunwaldzka 42, 14-100 Ostróda, Poland

**Keywords:** infectious bronchitis, chickens, vaccination, serological baseline values, efficacy under field conditions

## Abstract

Infectious bronchitis virus (IBV) is a highly infectious disease of chickens posing a major threat to poultry production worldwide. Due to genetic and antigenic variability, it is difficult to establish one universal protocol for vaccination against IB. The protectotype vaccination strategy, combining antigenically distinct vaccine strains, offers broader cross-protection than homologous IBV vaccination approaches. This field study aimed to establish serological baseline values in broiler chickens in a protectotype vaccination program using Avishield IB H-120 and Avishield IB GI-13, and to evaluate the program’s efficacy under field conditions on commercial farms where heterologous IBV genotypes were detected. The study was conducted on 25 broiler farms in 2023–2024. Day-old chicks were vaccinated via coarse spray. Blood samples were taken from them at six weeks of age and analyzed using IDEXX and BioChek ELISAs, and cecal tonsils were tested by real-time RT-PCR. Production performance was assessed using the European production efficiency factor (EPEF), with a cut-off value of 360. No IB-related clinical signs were reported, and the mean EPEF of the included farms was 408.7. The detected IBV genotypes were Mass + 793B, 793B, and 793B + VAR2. The presence of heterologous VAR2 did not negatively impact flock health or performance. Preliminary serological baseline values for vaccinated, non-VAR2 farms were established at 891–1332 (in chicks tested with the IDEXX ELISA) and 4193–5204 (in those tested with the BioChek assay). The H-120 + GI-13 program demonstrated high field efficacy, reliable serological profiles, and cross-protection in the presence of heterologous IBV. Continuous monitoring of IBV genotypes remains essential.

## Simple Summary

Infectious bronchitis (IB) is a highly infectious, viral disease in chickens contributing to very high production losses in the poultry industry worldwide. Specific prophylaxis is widely applied in chickens in order to control the damage caused by this disease. Despite the fact that numerous companies produce specific IBV vaccines, there are scarce field efficacy evaluation data available for them. The data by which to interpret serological results after the use of a given vaccination protocol against IB are also poor. Acknowledging the sparsity of data, this study sought to establish standards for serological evaluation (calculation of serological baseline values) of a vaccination program of broiler chickens against IB using Avishield IB H-120 and GI-13 vaccines. An additional goal of this study was to verify the efficacy of this program under field conditions. The presented results show that the vaccination protocol applied was very efficient in protecting flocks from clinical outbreaks after heterologous IBV infection. They also provide serological baseline values for the two most commonly used serological tests.

## 1. Introduction

Infectious bronchitis (IB) is a highly contagious viral disease in chicken caused by infectious bronchitis virus (IBV), a gamma coronavirus. IBV is characterized by a high mutation and recombination rate, particularly within the S1 subunit of the spike glycoprotein. Genetic and antigenic variation within S1 contributes to altered tissue tropism of IBV, as well as to its pathogenicity, immune evasion, and post-vaccination infectivity in breakthrough cases [1,2,3,4,5,6,7]. Due to the extensive diversity of circulating IBV strains, it is challenging to design a single universal vaccination program. As in other poultry diseases, the primary principle of IBV vaccination is to match the circulating field virus as closely as possible with the homologous vaccine strain which is given. In broiler production, especially in high-density poultry production areas, in theory the dominant field genotype in one production cycle may differ from that in the next, making rigidly homologous approaches impractical [7,8,9,10,11,12].

Cross-protection against heterologous IBV strains has been extensively documented. For example, vaccination of day-old chicks with either Mass-like or 793B vaccine strains can induce 100% protection against homologous challenge and at the same time, these vaccinations also significantly reduce ciliostasis in tracheal epithelial cells following heterologous IBV challenge with strains such as D207, D1466, QX-like, and others [13,14]. Ciliostasis is a well-known consequence of IBV infection, resulting from the cytopathic effect of IBV on the epithelial cells in the upper respiratory tract of chickens [13]. These findings affirm the practical value of the widely adopted protectotype concept in IBV immunoprophylaxis, in which combinations of genetically and antigenically distinct vaccine strains broaden the spectrum of cross-protection compared to monovalent vaccination [13]. Protectotype-based programs enable a degree of standardization across broiler farms, regardless of the specific circulating field strain’s similarity to or difference from the vaccines. This strategy is widely used globally in broilers, layers, and broiler breeders.

One of the first studies describing the IB protectotype vaccination strategy was published by Cook et al. [13], demonstrating that combined Ma5 (Mass genotype) and 4/91 (793B genotype) vaccination provides markedly improved protection against heterologous challenge. More recently, Kutle et al. [15] showed that even without the use of a QX-like vaccine strain, protectotype programs achieved approximately 70–75% protection against QX-like challenge. However, most of the studies regarding protectotype vaccination strategy against IBV were performed under laboratory conditions with the eye-drop vaccination technique. Data are missing on the efficacy of this vaccination strategy under field conditions where mass-application techniques (especially vaccination via spray) are applied. Another major advantage of standardized IB protectotype vaccination is the possibility of establishing reliable serological baseline values in IB-vaccinated chicken flocks. These reference titers allow accurate assessment of flock status. For example, unexpectedly high titers may indicate field exposure, whereas unusually low titers may indicate poor vaccination quality. Unfortunately, the available publications on IBV protectotype vaccination programs lack enough quantitative serological data for useable reference values. Against this background, a field study was carried out in order to establish standards for serological evaluation (through calculation of serological baseline values) of a program of vaccination of broiler chickens against IB, using Avishield IB H-120 (of Mass genotype) and Avishield IB GI-13 (of 793B genotype) preparations. Additionally, a goal of this study was to verify the efficacy of this vaccination program under field conditions—based on serological and clinical evaluation of flocks in which genotypes other than Mass and 793B IBV had been detected.

## 2. Materials and Methods

### 2.1. Experimental Design

The experiment was conducted under field conditions on commercial broiler farms between 2023 and 2024. A total of 25 farms were tested. These farms were under the veterinary care of 8 independent practices. In order to standardize the farms tested in this study, participating vets were asked to comply with the following criteria for the farms: max no of chicken houses = 4; max capacity = 30,000–50,000 chickens per one chicken house; farms with very high biosecurity standards. Vaccination with Avishield IB H-120 and Avishield IB GI-13 (Dechra Veterinary Products, Warsaw, Poland) was performed via coarse spray on the day of hatching on the farm in accordance with generally accepted good veterinary practice. Both vaccines were dissolved in water and given to birds simultaneously. The amount of water used was based on the flock size. An adequate number of doses of the appropriate vaccine for the flock size was dissolved in water and administered via coarse spray when the chicks were still held in transport boxes. The vaccine solution was used within a maximum of 1–2 h after reconstitution. After the vaccinations, the birds were kept in boxes for an additional 10 to 15 min before they were returned to the litter. More details describing the vaccination technical approach can be found in Supplementary Material S1.

In cases where there was more than one chicken house on a farm, all of them were vaccinated with the same anti-IB protocol, but only one chicken house was selected for further sampling.

At the end of the production cycle (at approximately 6 weeks of life) blood samples and cecal tonsils (CT) were collected from the birds. The number of samples collected per flock was 23 for blood and 5 for CT. In order to collect the CT, the birds were euthanized by the veterinarians by cervical dislocation. A simple summary of the experimental design for an individual farm is presented in Table 1. Blood samples were centrifuged in order to obtain serum samples, which were further processed for serological evaluation. CT were used for PCR analysis. Flock production results were made available from all the test farms. Additionally, all veterinarians were obliged to report clinical observations from the enrolled farms and, in some cases, the results of laboratory tests on research flock birds.

All vets participating in the study were asked about their coarse spray vaccination technique (especially the type of sprayer used, water quality, and water temperature). The results of this survey and its analysis can be found in Supplementary Material S1.

### 2.2. Serology

Infectious bronchitis serological evaluations in six-week-old birds were performed with two commercial ELISA kits (from IDEXX, Westbrook, ME, USA and BioChek, Reeuwijk, The Netherlands). Successive steps of the ELISA tests were performed according to the manufacturers’ recommendations. The ELISAs were carried out with the use of the Eppendorf epMotion 5075 LH automated pipetting station (Eppendorf, Hamburg, Germany), BioTek ELx405 automatic plate washer (BioTek, Winooski, VT, USA) and BioTek ELx800 plate reader. Internal positive controls (GD Animal Health, The Netherlands) were incorporated to validate the test results. For individual samples the S/P (sample to positive) values were calculated based on the optical densities (OD) of the sample and positive and negative controls. S/P values were used to calculate the antibody titers for individual samples in the software provided by IDEXX and BioChek for the interpretation of their ELISA tests. The kit manufacturers’ standard cut-off values were applied in order to interpret the samples as negative or positive. The individual titers of IB antibodies for each sample were used to express the mean geometric titer (Gmean) for the evaluated sample batch. The Gmean value for IDEXX and the Gmean value for BioChek were used to find the mean Gmean value for each veterinary practice. Additionally minimum and maximum titers were recorded, as well as the mean number of positive and negative samples per sample batch for individual veterinary practices. For farms with the same IBV genotype scenarios, kit-specific Gmean values were used to express mean Gmean values. Similarly to how minimum and maximum titers and the mean number of positive and negative samples per sample batch were found for veterinary practices, they also were for each IBV genotype scenario.

### 2.3. Detection and Differentiation of IBV by Real Time RT-PCR Evaluation

After collection, CT samples for PCR were suspended in physiological fluid and partially homogenized using glass beads. Total viral genetic material from the prepared samples was isolated using a commercial MagMAX™ CORE Nucleic Acid Purification Kit (Thermo Fisher Scientific, Waltham, MA, USA) and a KingFisher™ Duo Prime Purification System (Thermo Fisher Scientific, USA). The resulting product was used to detect the presence of IBV RNA by real-time RT-PCR. This was performed using commercial kits (Kylt, Höltinghausen, Germany) designed for the detection and differentiation of different IBV genotypes (Mass, 793B, D274, QX, D1466, IB80, and VAR2) using specific primers. Real-time RT-PCR reactions were performed according to the protocol provided by the manufacturer using a BioRad 7500 CFX96 Real Time System thermocycler (BioRad, Hercules, CA, USA). Fluorescence results were read using dedicated CFX Manager Dx software (BioRad CFX Maestro 2.3, BioRad, USA).

### 2.4. Production Results Calculation

The European production efficiency factor (EPEF) was calculated with the use of the following formula: EPEF = (survival rate (%) × final body weight (kg))/(age at slaughter × FCR (kg/kg)) × 100. An EPEF of 360 was used as a cut-off value. This cut-off value was proposed by the representative of one of the biggest Polish poultry integration in a personal communication. Production cycles with EPEF below 360 were not considered for further evaluation if the factor behind poor EPEF was not related directly to IB. If so, the data from those production cycles was discarded.

### 2.5. Statistical Analysis

The results of serological examination were analyzed statistically with one-way ANOVA (Statistica 13.1, StatSoft, Tulsa, OK, USA). The normality of residuals was assessed using the Shapiro–Wilk test, and the homogeneity of variances was verified by Levene’s test. As assumptions were met (*p* > 0.05), Tukey’s HSD test was applied for post hoc comparisons. Differences were considered significant at *p* ≤ 0.05.

## 3. Results

### 3.1. Clinical Observations and EPEF

None of the practitioners participating in this study reported any IB-related clinical symptoms. In most of the cases, the EPEF value was above 360. We have reported four cases/farms where EPEF value was below 360 (one case of Mareks disease outbreak, two cases of slow growing broilers and one unidentified cause but not related to IB). The mean EPEF value for the remaining farms (*n* = 21) was 408.7.

### 3.2. RT-PCR, Serology and EPEF

RT-PCR, serological and production results combined are presented in Table 2. Three different IBV-genotype scenarios were detected on test farms: Mass + 793B (*n* = 3), 793B alone (*n* = 10/14, where four cases were discarded due to an EPEF below 360) and 793B + VAR2 (*n* = 8). Although no additional sequencing of IBV was performed, every time a Mass or 793B genotype was detected it was considered to be of vaccine origin. This interpretation accords with the absence of IB-related clinical symptoms reported by the veterinarians.

Different Gmean titers were detected for test farm groups with different IBV-genotype scenarios, with the highest Gmean titer for the farm group where VAR2 was detected, although these differences were not statistically significant. No statistical differences were detected for mean EPEF values between farm groups with different IBV-genotype scenarios. For the 793B-only farm group, two mean EPEF values are indicated: one with all 14 cases (EPEF = 390) and the second after discarding four cases (EPEF = 405) with EPEF below 360. In this group serological results were calculated only for the farms with EPEF > 360.

Another combination of serological results and the IBV genotypes detected on test farms, especially with regard to Mass-genotype detection, is presented and analyzed in Supplementary Material S2.

### 3.3. Serological Results and Veterinary Practices

The Gmean titers recorded in the IDEXX and BioChek ELISA tests for different veterinary practices are combined in Table 3. Statistical differences were found in Gmean titers in the BioChek IBV ELISA test between different practices. To some extent, these results are also analyzed in Supplementary Material S1.

## 4. Discussion

Infectious bronchitis is one of the most important and economically significant diseases in chicken flocks, contributing every year to significant production losses [16,17]. The main strategies for preventing IB in poultry flocks are biosecurity, the appropriate management for the type of production, and specific immunoprophylaxis [1,3,8,9,10,12]. It has been shown many times in the past that the strategy of protectotype immunization of birds against IB brings very good and measurable results, reducing production losses by minimizing the risk of clinical occurrence of this disease in the flock [13,14,15,18,19,20]. From the present study it can be concluded that the applied vaccination program (H120 + GI-13) gave reliable efficacy in protection against IB. The conclusion rests on several facts, one fundamental one being that none of the veterinarians participating in the study reported health problems or clinical symptoms that could be associated with IB on the test farms. On all farms but the four described in Section 3, very satisfactory production results were recorded, which calculated to the overall mean EPEF of 408.7. In this study we have decided to discard results from farms when the EPEF did not meet production criteria for profitability. We have assumed that in this type of situation (if they are confirmed not to be caused by IB directly) there might have been other infection agents, also immunosuppressive, which could influence the results obtained in the laboratory analysis or there are other factors related, for example, production technology, which might have significantly distorted the picture of the obtained results.

The results for the group of farms on which the presence of the heterologous VAR2 IBV strain was recorded provided interesting data. Although a foreign IBV was present there, this did not negatively affect the clinical flock situation, which merited no reports of symptoms from veterinarians, nor the production results obtained on these farms. These businesses’ mean EPEF value was 416.00 and was higher than the overall mean for all farms. The highest mean IBV antibody titers were recorded in this group, which can be explained by additional stimulation of the birds’ immune systems by a foreign IBV strain. Taking into account the predefined criteria of the experiment, the serological results from this group were not included in the calculations of final reference values for the serological evaluation of the applied vaccination program.

Considering the positive VAR2 results, two scenarios should be kept in mind. The first would be the one of a field origin for this VAR2; then the applied vaccination program should be evaluated as highly effective in protection against VAR2 in broiler flocks. The second scenario, on the other hand, would assume that the origin of this virus is as a vaccine contaminant. In Poland, a commercial IB vaccine is available that contains the VAR2 strain. From unpublished data it is known that for Polish broilers, this is the vaccine most frequently purchased and used by veterinarians. Based on field observations, a very probable carry-over effect comes with this vaccine’s use, and the possibility of detection of the vaccine virus in subsequent production cycles is high. However, according to the interviews conducted among the veterinarians participating in the experiment, it turned out that the VAR2 strain had never been used in the past to immunize birds on any of the farms.

Ultimately, it is likely that the applied vaccination program effectively protected against uncontrolled replication of the heterologous VAR2 virus (regardless of its origin) in the flock. Incidents of infection with the VAR2 virus may have contributed to an increase in the mean geometric antibody titer against IBV in the flocks, and a real assessment of the impact of widespread use of the VAR2 vaccine on its rolling under field conditions, the potential risk of reversion to a virulent form, and the current epidemiological situation of VAR2 strains in general require urgent additional studies. On the other hand increased GMT in VAR2 positive farms could also be the result of different vaccination techniques (see Supplementary Material S1 for more details) or homologous Mass or GI-13 infection in this flock (discussed later).

Ultimately, the baseline values for serological evaluation of the vaccination program utilizing the H120 + GI-13 strains were calculated only from the mean minimal and maximal values from the two groups of farms where only genetic material homologous to the vaccine viruses was amplified. This criterion produced estimated preliminary baseline values of 891–1332 in the case of the IDEXX test and 4193–5204 in the case of the BioChek test. These values correspond with those from field observations for farms immunized with similar vaccination programs, as well as with the recommended Gmean titer values published online by the BioChek manufacturer for the Mass + 793B vaccination program [21]. In this study we decided to use both IDEXX and BioChek ELISA tests simultaneously due to their equally frequent use in the serological monitoring of IB in chickens by veterinarians in Poland.

What should be emphasized with regard to the presented data is that, as mentioned previously, no additional sequencing of IBV was performed under this study. We assumed that every time a Mass or 793B genotype was detected, it was of vaccine origin. To some extent this interpretation is in line with the fact that there have been no IB related clinical symptoms reported by vet practitioners; however, we cannot exclude the possibility of homologous IBV infection on the farm, which is a major limitation of this work. The second biggest limitation of this study was the small size of the analyzed groups; therefore, the results obtained should be considered as a reference point rather than universal reference values. Additionally, possible discrepancies in the obtained serological results may have been caused by the practices used by a given veterinarian during bird vaccination. From our own observations it seems that these results depend primarily on the care taken in preparation of the water in the vaccine solution and on the quality of this water itself (see Supplementary Material S1 for more details). Given that serum antibody levels correlate to some extent with the level of post-vaccination protection against IBV [22], it seems logical that higher levels represent a higher level of protection of the flock against the disease. From this point of view, every effort should be made to ensure that the vaccination procedure is performed according to the highest standards, based on available scientific and practical knowledge. Additionally, serological results may differ from the baseline values in the situation of the appearance of a heterologous IBV on the farm, regardless of its origin (vaccine or field origin).

## 5. Conclusions

The combined H120 + GI-13 vaccination program demonstrated putative effectiveness under field conditions against IBV, effectively prevented the clinical disease, maintained high production performance of chickens and provided cross-protection in the presence of heterologous IBV genotypes such as VAR2. Reliable serological baseline values were established for flocks immunized with the IDEXX and the BioChek ELISAs, enabling more precise interpretation of post-vaccination serology under field conditions. Given the frequent detection of heterologous IBV genotypes in commercial broiler production, continued monitoring of genotype prevalence, the dynamics of vaccine virus rolling, and possible reversion to virulence (particularly of VAR2 vaccine strains) is of great importance.

## Figures and Tables

**Table 1:** Simple summary of the experimental design for an individual farm with the indication of the birds’ age at the time of vaccination and sample collection.

Bird Age	Procedure
Day of hatching	Coarse spray vaccination in transport boxes on the farm against IB with the use of H-120 and GI-13 antigens
Approx. 6 weeks	Collecting of blood samples (*n* = 23) for serological evaluation of IB antibody titers with IDEXX and BioChek ELISA tests
Approx. 6 weeks	Collecting of CT samples (*n* = 5) for the RT-PCR evaluation of different IBV genotypes

**Table 2:** Gmean titer evaluated with the use of IDEXX and BioChek ELISA tests for chicken farms with different IBV-genotype scenarios together with mean production results presented as mean EPEF value. GMean titer was calculated based on the Gmean titer values for individual farms with the same IBV-genotype scenario identified. Max and Min represent minimum and maximum values recorded in IDEXX and BioChek for individual samples from the same group of results (groups determined based on PCR results). Number POS and Number NEG represent mean number of positive and negative samples (per sample batch).

IBV Genotypes Identified	Number of Cases	Gmean Titer IDEXX	Max IDEXX	Min IDEXX	Number POS IDEXX	Number NEG IDEXX	Gmean Titer BioChek	Max BioChek	Min BioChek	Number POS BioChek	Number NEG BioChek	European Production Efficiency Factor (EPEF)
793B + Mass	3	891.67 ^A^ *	6351.67	72.33	18.67	4.33	4193.33 ^A^	11,558.33	1101.67	22.67	0.33	416 ^A^
793B	14	1332.86 ^A^	5009.64	323.00	18.21	4.86	5204.57 ^A^	12,420.00	1438.50	22.71	0.36	390/405 ^A^
793B + VAR2	8	1553.00 ^A^	3672.88	1722.25	19.75	3.25	6601.75 ^A^	13,973.50	2387.63	22.75	0.25	416 ^A^

* A—values for a given parameter with different superscript letters represent statistical difference between groups.

**Table 3:** Mean GMT evaluated with the use of IDEXX and BioChek ELISA tests for chicken farms under different DVM supervision. Mean GMT was calculated based on the GMT values for individual farms supervised by one DVM. Max and Min represent minimal and maximal values recorded on IDEXX and BioChek for individual samples from the same group (groups determined based on the supervising DVM). No POS and No NEG represent mean number of positive and negative samples (per each sample batch).

DVM	Gmean Titer IDEXX	Inter-Group Difference	Max IDEXX	Min IDEXX	Number POS IDEXX	Number NEG IDEXX	Gmean Titer BioChek	Inter-Group Difference	Max BioChek	Min BioChek	Number POS BioChek	Number NEG BioChek
1	1091.00	A *	3816.00	233.00	20.00	3.00	3224.00	B	6712.00	1459.00	23.00	0.00
2	599.83	A	3736.00	58.67	15.17	7.67	4021.50	B	9556.00	752.50	22.00	0.83
3	888.50	A	6141.00	113.00	22.00	1.00	4285.50	B	12,910.00	1484.00	23.00	0.00
4	782.67	A	3995.67	110.00	16.00	7.00	4594.33	B	12,824.33	1080.67	23.00	0.00
5	1548.86	A	4781.71	1887.00	20.43	2.86	5715.29	AB	14,151.00	1709.14	23.14	0.14
6	1572.50	A	4990.00	396.00	20.00	3.00	7281.00	A	15,355.50	2259.50	23.00	0.00
7	3076.00	A	7013.00	991.67	22.67	0.33	8059.00	A	15,321.33	3166.67	22.67	0.33
8	1729.00	A	6706.00	503.00	23.00	0.00	9805.00	A	18,496.00	5132.00	23.00	0.00

* A, B—values for a given parameter with different letters represent statistical difference between groups.

## Data Availability

Data are contained within the article. The datasets used and/or analyzed during the current study are available from the corresponding author on reasonable request.

## Supplementary Materials

The following supporting information can be downloaded at: https://www.mdpi.com/article/10.3390/ani16050807/s1, Supplementary Material S1. The impact of IB vaccination techniques (type of sprayer, water quality) on serological results; Supplementary Material S2. Hypothesis on Mass genotype detection in vaccinated flocks of chickens. Refs. [23,24] are cited in the Supplementary Materials.

## Abbreviations

The following abbreviations are used in this manuscript:

CT	Cecal Tonsils
DVM	Doctor of Veterinary Medicine
ELISA	Enzyme Linked Immunosorbent Assay
EPEF	European Production Efficiency Factor
FCR	Feed Conversion Ratio
GMT	Geometric Mean Titer
IB	Infection Bronchitis
IBV	Infectious Bronchitis Virus
No NEG	Number of Negative Samples
No POS	Number of Positive Samples
RT-PCR	Reverse-Transcriptase Polymerase Chain Reaction
