# Supplementary material for: Evaluation of the Serological Baseline Values of Broiler Chickens Jointly Vaccinated with Infectious Bronchitis H + 120 and GI-13 Preparations Under Field Conditions
Source: Animals (Basel). 2026 Mar 5;16(5):807. doi: 10.3390/ani16050807 (PMC12984738; doi:10.3390/ani16050807)
Supplement: Supplementary file 1 [file animals-16-00807-s001.zip › animals-4150669-supplementary.pdf]

Supplementary material S1

After the study was conducted the vets where asked the following questions regarding the vaccination technique:

1) Type of sprayer used to vaccinate the birds?
2) Which nozzle was used for the vaccination?
3) What was the water source?
4) What was the water temperature for the vaccination preparation and during vaccination itself?

Six (DVM 1-5 and 7) out of eight vets used the same sprayer (Birchmeier) with most of them (five out of six) using the red nozzle. In Supplementary Table S1 below the results of this survey can be found, where six vets (using Birchmeier sprayer) are grouped according to their average anty – IBV antibodies GMT (based on Biochek results; from the lowest GMT to the highest). Furthermore you can find the water quality and temperature differences between the vets with the lowest (tap water at room temperature over summer) and highest (water from reverse osmosis, as cold as possible) GMT results. It has been demonstrated earlier that with every +1 degree Celcius of water temperature (ranging between 6 and 20 degrees) for IB vaccination there was a decrease by 3.2% of antibody production levels after the vaccination [23].

Supplementary Table S1. Results of the survey conducted with the vets. Six out of eight vets using Birchmeier sprayer are grouped accoring to the average anty-IBV GMT values recorded on Biochek IBV ELISA test. Differences between water quality and its temperature for vaccination are highlighted. Subscription letters A-B indicate statistical differences for GMT values on Biochek test.

	Lab Results		Spray characteristic				
DVM	**Mean GMT IDEXX**	**Mean GMT BioChek**	**Sprayer**	**Nozzle**	**Water quality**	**Water temperature**	**Statistics for Biochek rsults**
1	**1091,00**	**3224,00**	Birchmeier	red	tap water with AviBlue	room (summer)	B
2	**599,83**	**4021,50**	Birchmeier	yellow	destilled	room (autumn)	B
3	**888,50**	**4285,50**	Birchmeier	red	demineralized	room (winter)	B
4	**782,67**	**4594,33**	Birchmeier	red	mineral	room (summer)	B
5	**1548,86**	**5715,29**	Birchmeier	red	destilled	cold	AB
7	**3076,00**	**8059,00**	Birchmeier	red	reverse osmosis	as cold as possible	A

References

23. De Wit, J. J., Swart, W. A. J. M., Fabri, T. H. F. Efficacy of infectious bronchitis virus vaccinations in the field: association between the α-IBV IgM response, protection and vaccine application parameters. *Avian Pathol.*, 2010, 39, 123–131.

Supplementary material S2

In Supplementary Table S2 below an indication of three cases where Mass genotype was detected can be found. As it turned out Mass was detected in case of three vets (DVM 1, 3 and 5) who were grouped within the vets with lower GMT values. As further highlighted Mass was detected on farms with GMT values below the average GMT values recorded for a given vet. The hypothesis behind this is that in these cases

(Mass detection cases) the technique of the vaccination was poor which would explain low GMT values. Poor technique could be a result of low water quality or its high temperature (see additional material 1 for more details). Additionally it has been sugested earlier that IBV 793B vaccine strains can inhibit the replication of Mass vaccine strains [24]. In the case of poor vaccination technique it could hypothetized that 793B virus was unable to replicate in target cells sufficiently effective to the level where it is capable of inhibiting the Mass replication. Therefore Mass was able to replicate to a higher titer and therefore it could still be detected at approx. 6 weeks of a bird's life. Although this hypothesis sound justified not only by the results of this study but also field observations (data not published), it needs further studies for confirmation purposes.

Lab Results												
DVM	**Mean GMT IDEXX**	**Statistics**	Max IDEXX	Min IDEXX	No POS IDEXX	No NEG IDEXX	**Mean GMT BioChek**	**Statistics**	Max BioChek	Min BioChek	No POS BioChek	No NEG BioChek
1	**1091,00**	A	3816,00	233,00	20,00	3,00	**3224,00**	B	6712,00	1459,00	23,00	0,00
2	**599,83**	A	3736,00	58,67	15,17	7,67	**4021,50**	B	9556,00	752,50	22,00	0,83
3	**888,50**	A	4578,50	61,50	18,00	5,00	**4285,50**	B	11797,00	1107,00	22,50	0,50
4	**782,67**	A	3995,67	110,00	16,00	7,00	**4594,33**	B	12824,33	1080,67	23,00	0,00
5	**1548,86**	A	4781,71	1887,00	20,43	2,86	**5715,29**	AB	14151,00	1709,14	23,14	0,14
6	**1572,50**	A	4990,00	396,00	20,00	3,00	**7281,00**	AB	15355,50	2259,50	23,00	0,00
7	**3076,00**	A	7013,00	991,67	22,67	0,33	**8059,00**	A	15321,33	3166,67	22,67	0,33
8	**1729,00**	A	6706,00	503,00	23,00	0,00	**9805,00**	A	18496,00	5132,00	23,00	0,00

Mass detected at GMT:
1 – 680
3 – 528
5 – 1 249

Mass detected at GMT:
1 – 3 505
3 – 3 474
5 – 5 097

Supplementary Table S2. Three cases of Mass genotype detection are highlighted (Vets no 1, 3 and 5). GMT values recorded on IDEXX (left side below the table) and Biochek (right side, below the table) for these three cases are indicated. Subscription letters A-B represent statistical differences between GMT values on IDEXX or Biochek IBV ELISA tests.

References

24. Tucciarone CM, Franzo G, Lupini C, Alejo CT, Listorti V, Mescolini G, Brandão PE, Martini M, Catelli E, Cecchinato M. Avian Metapneumovirus circulation in Italian broiler farms. Poult Sci. 2018, 97, 503-509.
